# The novel ABC transporter ABCH1 is a potential target for RNAi-based insect pest control and resistance management

**DOI:** 10.1038/srep13728

**Published:** 2015-09-03

**Authors:** Zhaojiang Guo, Shi Kang, Xun Zhu, Jixing Xia, Qingjun Wu, Shaoli Wang, Wen Xie, Youjun Zhang

**Affiliations:** 1 Department of Plant Protection, Institute of Vegetables and Flowers, Chinese Academy of Agricultural Sciences, Beijing 100081, China

## Abstract

Insect pests cause serious crop damage and develop high-level resistance to chemical insecticides and *Bacillus thuringiensis* (Bt) insecticidal Cry toxins. A new promising approach for controlling them and overcoming this resistance is RNA interference (RNAi). The RNAi-based insect control strategy depends on the selection of suitable target genes. In this study, we cloned and characterized a novel ABC transporter gene *PxABCH1* in diamondback moth, *Plutella xylostella* (L.). Phylogenetic analysis showed that *PxABCH1* is closely related to ABCA and ABCG subfamily members. Spatial-temporal expression detection revealed that *PxABCH1* was expressed in all tissues and developmental stages, and highest expressed in head and male adult. Midgut sequence variation and expression analyses of *PxABCH1* in all the susceptible and Bt-resistant *P. xylostella* strains and the functional analysis by sublethal RNAi demonstrated that Cry1Ac resistance was independent of this gene. Silencing of *PxABCH1* by a relatively high dose of dsRNA dramatically reduced its expression and resulted in larval and pupal lethal phenotypes in both susceptible and Cry1Ac-resistant *P. xylostella* strains. To our knowledge, this study provides the first insight into ABCH1 in lepidopterans and reveals it as an excellent target for RNAi-based insect pest control and resistance management.

Herbivorous insect pests cause severe crop damage and economic losses worldwide. Although chemical insecticides remain the major tool used to control insect pests, the chemicals can be hazardous to the environment and human health. A valuable alternative to chemical insecticides is the planting of transgenic crops that produce the insecticidal Cry toxins of *Bacillus thuringiensis* (Bt crops), and more than 75 million hectares of Bt crops were planted worldwide in 2013^1^. In recent years, however, insect resistance to Bt crops has developed rapidly^2^. As a consequence, there is an urgent need for a novel control method that can replace or be used in combination with Bt crops as part of the integrated pest management (IPM) strategies.

RNA interference (RNAi) is a fascinating gene regulation mechanism that is ubiquitous and evolutionarily conserved in many eukaryotes including insects^3^. In RNAi, the 21- to 23-nt short interfering RNAs (siRNAs) generated from long double-stranded RNAs (dsRNAs) can cleave complementary messenger RNA (mRNA) and mediate sequence-specific post-transcriptional gene silencing^4^. Thus far, RNAi has become an important technology for the study of gene function in insects, especially in non-model insects^5^,^6^. Moreover, RNAi-mediated insect pest management strategies have been developed in the form of both species-specific biopesticides and as next-generation transgenic plants^7^,^8^,^9^,^10^,^11^.

The diamondback moth, *Plutella xylostella* (L.) (Lepidoptera: Plutellidae), is a cosmopolitan and devastating pest of cruciferous crops. Globally, direct losses and control costs for this pest are now estimated to be US $ 4–5 billion annually^12^. Until now, the application of chemical insecticides still remains the major strategy for controlling *P. xylostella* because these chemicals are easy to apply and have been effective. Continuous insecticide application, however, has resulted in *P. xylostella* developing high levels of resistance to almost all the synthetic and biological insecticides (http://www.irac-online.org/pests/plutella-xylostella/). In particular, *P. xylostella* was the first documented insect pest to have developed Bt resistance in the field^13^. RNAi has been recently achieved in *P. xylostella* by both dsRNA injection and oral delivery^14^,^15^,^16^,^17^, suggesting that RNAi technology is very feasible for the control of *P. xylostella*. Noteworthy, the use of an RNAi-based strategy for control of *P. xylostella* relies mostly on identifying suitable target genes to silence.

The ATP-binding cassette (ABC) transporter superfamily is one of the largest groups of membrane proteins in all kingdoms of life, most of which participate in ATP-dependent transport of a wide array of substrates across cellular membranes^18^. The ABC transporters have two highly conserved core function domains including the nucleotide binding domain (NBD), which is located in the cytoplasm and can utilize ATP to provide energy, and the transmembrane domain (TMD), which is embedded in the lipid bilayer and is involved in the translocation of specific substrates^19^. Full transporters consist of two NBDs and two TMDs, while half transporters have only one NBD and one TMD, which must form homo- or hetero-dimers to be a functional unit^20^. Based on NBD sequence similarity, the ABC transporter superfamily can be divided into eight subfamilies (ABCA to ABCH)^21^.

The unique ABCH transporter subfamily was first discovered in the genome of the fruit fly *Drosophila melanogaster*^19^. ABCH transporter genes appear to be present in all insects, non-insect arthropods (such as the spider mite *Tetranychus urticae* and the waterflea *Daphnia pulex*), non-insect metazoas (including teleosts and the purple sea urchin *Strongylocentrotus purpuratus*), and non-insect protozoans (like the slime mould *Dictyostelium discoideum*), but they are absent from plants, worms, yeast, and mammals^21^,^22^,^23^,^24^,^25^,^26^,^27^,^28^,^29^,^30^,^31^,^32^,^33^. Like ABCG proteins, ABCH proteins are half transporters and have a reverse-domain arrangement, with the NBD at the N-terminus end and the TMD at the C-terminus.

Although ABCH transporters have been found in many species, the detailed studies about them still remain limited. A previous study showed that the knockout mutants and a knockdown RNAi line of an ABCH gene *CG9990* in *D. melanogaster* are both lethal^34^. Subsequently, a study reported the cloning and preliminarily characterization of the *DrABCH1* gene (orthologous gene of *CG9990*) in zebrafish *D. rerio*^30^. Recently, an excellent study utilized RNAi to demonstrate that knockdown of the *TcABCH-9C* (orthologous gene of *CG9990*) can result in the larval desiccation phenotype and negative effects on adult egg-laying and egg-development that is similar to knockdown of *TcABCG-4C* in *Tribolium castaneum*, suggesting that TcABCH-9C might also act as transporters of cuticular lipids just like TcABCG-4C in *T. castaneum*^34^. Since that these ABCH transporters only exist in invertebrate (except some teleosts like zebrafish) and silencing of their expression can induce lethal phenotype in insects, they might be used as suitable targets for RNAi-based insect pest control^35^,^36^. Moreover, given that the role of ABC transporters in chemical insecticide and Bt bioinsecticide resistance is well documented in the literature^36^,^37^ and elevated levels of ABCH transcripts have been reported in two insecticide-resistant arthropods including *P. xylostella*^26^ and *T. urticae*^37^, it is deserves to be seen whether ABCH transporters are associated with insecticide resistance in insects and it also deserves to be determined whether ABCH transporters can be used as potential targets for RNAi-based control of these insecticide-resistant insects.

In our previous transcriptome and RNA-seq studies, we found multiple ABC transporter genes were differentially expressed in the midgut tissue between Cry1Ac-susceptible and -resistant *P. xylostella* strains^38^,^39^. In the current study, we found an ABCH gene when re-analyzing and re-annotating them, indicating it may correlate with Cry1Ac resistance in *P. xylostella*. Therefore, we cloned and characterized this novel ABCH subfamily gene (*PxABCH1*, orthologous gene of *CG9990*) in *P. xylostella*, however, our overall experimental results demonstrated that *PxABCH1* gene is actually independent of Cry1Ac resistance in *P. xylostella*, furthermore, our *in vivo* RNAi results revealed that remarkably suppression of *PxABCH1* expression by injection or oral delivery of a relatively high dose of dsRNA was lethal to larvae and pupae in both susceptible and Cry1Ac-resistant *P. xylostella* strains, and the caused larval desiccation phenotype (albeit unconspicuous) indicated that the PxABCH1 might also act as transporters of cuticular lipids like its ortholog TcABCH-9C in *T. castaneum*. The results demonstrated that the ABCH1 can serve as a valuable target for the novel RNAi-based insect pest control and resistance management strategy.

## Results

### Cloning and characterization of the *PxABCH1* gene

**Figure 1:**
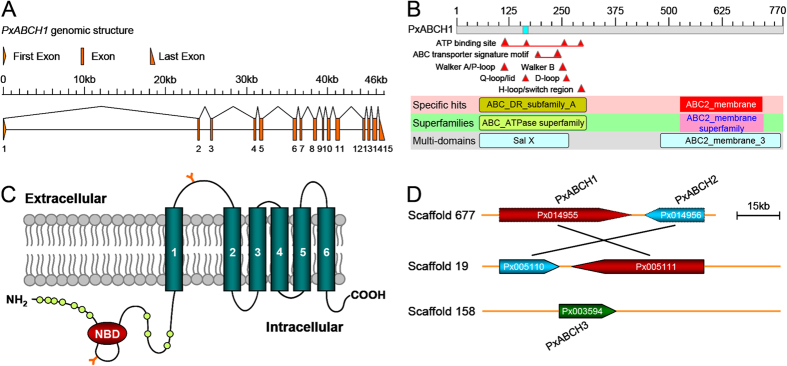
Gene and protein structure analyses of *PxABCH1* in *P. xylostella*. (**A**) Genomic structure of the *PxABCH1* gene. Brown boxes indicate the exons, and the spaces between two boxes are the introns. The figure is drawn to scale, and the scale bar is shown. (**B**) NCBI conserved domain database (CDD)-based annotation of the deduced PxABCH1 protein sequence. Based on its sequence, the PxABCH1 protein was identified as a member of the ABC transporter superfamily and contains Sal X and ABC2_membrane_3 multi-domain regions, which are characteristic of the ABC-type transport system. The specific hits incorrectly denoted it as a member of the ABCA subfamily probably because of the high sequence similarity between members from ABCH and ABCA transporter subfamilies. Segments of the sequence with low compositional complexity are colored blue. (**C**) Predicted protein topology of the PxABCH1 protein. The protein contains one NBD and one TMD with six membrane-spanning segments. “Y” represents predicted N-glycosylation sites, and circles indicate potential O-glycosylation sites. (**D**) Structure and location of the ABCH genes on *P. xylostella* genome scaffolds. Three ABCH genes including *PxABCH1* (Px014955 on scaffold_677 or Px005111 on scaffold_19), *PxABCH2* (Px014956 on scaffold_677 or Px005110 on scaffold_19) and *PxABCH3* (Px003594 on scaffold_158) are found in the *P. xylostella* genome database (DBM-DB: http://iae.fafu.edu.cn/DBM/index.php). The transcription orientations of these ABCH genes on the scaffolds are shown, and the *PxABCH1* and *PxABCH2* genes are tandemly arranged in a head-to-tail orientation.

To determine whether the *PxABCH1* gene is involved in *P. xylostella* Cry1Ac resistance, its full-length cDNA sequence (3556 bp) was cloned by 3′, 5′- rapid amplification of cDNA ends (RACE). It contains an open reading frame (ORF) of 2313 nucleotides that encode 770 amino acid residues, a 110-bp 5′-untranslated region (5′-UTR) and a 1133-bp 3′-UTR (Fig. S1). At the 3′ end of the *PxABCH1* cDNA sequence, a polyadenylation signal sequence AATAAA occurs upstream of the polyA tail. The full-length cDNA sequence has been deposited in GenBank under accession number KP260785. The complete genomic DNA (gDNA) sequence of *PxABCH1* (Gene ID: Px005111) was found by Blastn searching against the Diamondback Moth Genome Database (DBM-DB: http://iae.fafu.edu.cn/DBM/search.php) with the full-length cDNA sequence of *PxABCH1* as a query. Although the gDNA sequence of *PxABCH1* is complete, our bioinformatic analysis showed that its coding sequence (CDS) was incorrectly annotated in the genome database, and we corrected the CDS sequence of *PxABCH1* according to the cloned full-length cDNA sequence. Subsequent genomic structure analysis showed that the *PxABCH1* gene contains 15 exons including a small first exon (11 bp) and a large first intron (about 24 kb) (Fig. 1A). The calculated molecular weight (Mw) and isoelectric point (pI) of the predicted PxABCH1 protein are about 85.4 kDa and 6.52, respectively. The PxABCH1 protein has structural features that are characteristic of ABC transporters, and it is similar to those of ABCG subfamily members containing one cytoplasmic N-terminal nucleotide-binding domain (NBD) with several conserved motifs (Walker A, Walker B, ABC transporter signature motif, Q-loop, D-loop and H-loop) followed by one C-terminal transmembrane domain (TMD) with six transmembrane α-helix segments (Fig. 1B,C). Moreover, the PxABCH1 protein lacks a putative N-terminal signaling peptide like other ABC proteins, while it contains two potential N-glycosylation sites and 11 potential O-glycosylation sites (Fig. 1C), suggesting that it can be glycosylated. Indeed, the PxABCH1 ortholog in *D. melanogaster* (CG9990) is glycosylated as determined by SPEG-MS technique^40^. In addition, only three ABCH genes including *PxABCH1* (Px014955 on scaffold_677 or Px005111 on scaffold_19), *PxABCH2* (Px014956 on scaffold_677 or Px005110 on scaffold_19) and *PxABCH3* (Px003594 on scaffold_158) were found in the *P. xylostella* genome (Fig. 1D), which is also the case in most arthropods with the exception of *Lygus hesperus*, *T. urticae* and *D. pulex*^37^,^41^.

### Phylogenetic analysis of the *PxABCH1* gene

**Figure 2:**
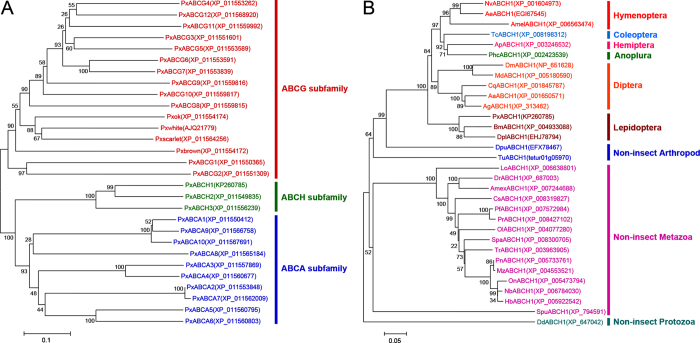
Phylogenetic analyses of the ABCH genes in *P. xylostella*(A) and the *ABCH1* genes in diverse species (B). (**A**) An unrooted neighbor-joining (NJ) tree was constructed on the basis of analyzing the amino acid sequences of the nucleotide binding domain (NBD) to show the phylogenetic relationships of ABCH subfamily genes with ABCA and ABCG subfamilies. Intriguingly, a previously undiscovered ABCG gene (*Pxbrown*) in the Diamondback moth Genome Database (DBM-DB, http://iae.fafu.edu.cn/DBM/index.php)^45^ has been found in the recent *P. xylostella* genome well re-annotated by NCBI (GenBank accession no. XM_011555870) and has been shown in the tree. (B) An unrooted neighbor-joining (NJ) tree was constructed on the basis of analyzing the full-length amino acid sequences to show the phylogenetic relationships of *ABCH1* genes from insect and non-insect species. Multiple amino acid sequence alignments for both of the phylogenetic analyses were generated by ClustalW, and the trees were subsequently created with MEGA 6.0. Bootstrap values expressed as percentages of 1000 replications are shown at branch points. GenBank accession numbers or Gene ID from other genome database sources are displayed within the tree and are indicated in parentheses. The scale bar indicates distance in number of substitutions per site. Abbreviations: **1. Hymenoptera** (**Amel**, *Apis mellifera*; **Nv**, *Nasonia vitripennis*; **Ae**, *Acromyrmex echinatior*); **2. Coleoptera** (**Tc**, *Tribolium castaneum*); **3. Hemiptera** (**Ap**, *Acyrthosiphon pisum*); **4. Anoplura** (**Phc**, *Pediculus humanus corporis*); **5. Diptera** (**Dm**, *Drosophila melanogaster*; **Md**, *Musca domestica*; **Aa**, *Aedes aegypti*; **Ag**, *Anopheles gambiae*; **Cq**, *Culex quinquefasciatus*); **6. Lepidoptera** (**Bm**, *Bombyx mori*; **Dpl**, *Danaus plexippus*; **Px**, *Plutella xylostella*); **7. Non-insect Arthropods** (**Tu**, *Tetranychus urticae*; **Dp**, *Daphnia pulex*). **8. Non-insect Metazoa** (**Dr**, *Danio rerio*; **Amex**, *Astyanax mexicanus*; **Pn**, *Pundamilia nyererei*; **Mz**, *Maylandia zebra*; **Spa**, *Stegastes partitus*; **Pf**, *Poecilia formosa*; **Tr**, *Takifugu rubripes*; **Pr**, *Poecilia reticulata*; **Ol**, *Oryzias latipes*; **Cs**, *Cynoglossus semilaevis*; **Nb**, *Neolamprologus brichardi*; **Hb**, *Haplochromis burtoni*; **Lo**, *Lepisosteus oculatus*; **On**, *Oreochromis niloticus*; **Spu**, *Strongylocentrotus purpuratus*). **9. Non-insect Prozotoa** (**Dd**, *Dictyostelium discoideum*).

The neighbor-joining (NJ) phylogenetic trees based on multiple sequence alignment of amino acid sequences of the identified NBDs for ABCA, ABCG and ABCH genes from *P. xylostella* or the full-length amino acid sequences for the *ABCH1* genes from diverse species were both constructed using MEGA 6.0 (Fig. 2). The unrooted tree for PxABCH genes showed that our cloned ABC transporter gene does belong to the ABCH subfamily, and despite the similarity in protein secondary structure between ABCH and ABCG members, the ABCH members are phylogenetically more closely related to the ABCA members than to the ABCG members (Fig. 2A). The unrooted tree for *ABCH1* genes displayed that the identified *ABCH1* genes are clearly grouped into one cluster for each insect order and for each group of non-insect species, suggesting *ABCH1* genes are evolutionarily conserved within each species. Although a recent study showed that teleosts seem to lack *ABCH1* except the zebrafish *D. rerio*^42^, we actually found *ABCH1* orthologous genes in recently available genome resources of many teleosts, and these orthologous genes form a clear cluster among the non-insect Metazoa in the tree (Fig. 2B). Moreover, *ABCH1* of the slime mould *D. discoideum* forms the most outside clan in the tree, suggesting it displays a specific evolutionary position between ABCH and other ABC transporter subfamilies (such as ABCA and ABCG) since that *D. discoideum* is a primitive eukaryotic organism just as described elsewhere^30^.

### Spatial-temporal expression pattern of the *PxABCH1* gene

**Figure 3:**
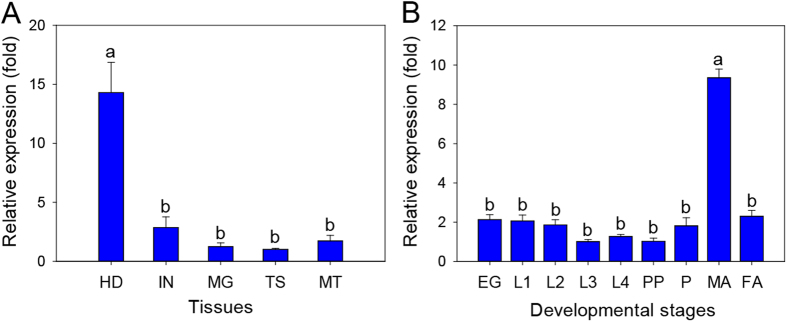
Spatial-temporal expression patterns of the *PxABCH1* gene as determined by qPCR in *P. xylostella*. (**A**) Relative expression levels of *PxABCH1* in the head (HD), integument (IN), midgut (MG), testis (TS) and Malpighian tubules (MT) of fourth-instar larvae. (**B**) Relative expression levels of *PxABCH1* in eggs (EG), first-instar larvae (L1), second-instar larvae (L2), third-instar larvae (L3), fourth-instar larvae (L4), prepupae (PP), pupae (P), and 1-day-old pre-copulatory male adults (MA) and female adults (FA). Expression of the *RPL32* gene was used as the internal reference gene to normalize data sets and to calculate expression differences. The relative expression level (fold) was calculated based on the value of the lowest expression detected (TS was used for tissues, and L3 was used for developmental stages), which was assigned an arbitrary value of 1. Values shown in the figures are means and standard errors. Different letters indicate significant differences among tissues or developmental stages based on three biological replications and four technical repeats (P < 0.05; Holm-Sidak’s test; n = 3).

To analyze the spatial expression patterns of *PxABCH1* in *P. xylostella*, we monitored its expression in five selected tissues from fourth-instar *P. xylostella* larvae using qPCR method (Fig. 3A). The qPCR analysis showed that *PxABCH1* was differentially expressed in the five tested tissues (P < 0.05; Holm-Sidak’s test; n = 3), and its expression was much higher in the head than in the other tissues.

We also used qPCR to assess the temporal expression pattern of *PxABCH1* in all the developmental stages of *P. xylostella* (Fig. 3B). We found that *PxABCH1* was expressed in all the developmental stages, but expression differed among the stages (P < 0.05; Holm-Sidak’s test; n = 3), and the *PxABCH1* expression was higher in male adults than in the other developmental stages (Fig. 3B).

### Relationship between the *PxABCH1* gene and Cry1Ac resistance in *P. xylostella*

A recent study showed that a mutated midgut ABCC transporter gene (*ABCC2*) is linked to Bt Cry1Ac resistance in *P. xylostella*^43^, and we also found that down-regulation of multiple ABC transporter genes (*ABCC2*, *ABCC3* and *ABCG1*) was associated with *P. xylostella* Cry1Ac resistance^44^,^45^. Therefore, we investigated the relationship between the *PxABCH1* gene and Cry1Ac resistance in *P. xylostella*. We first cloned the full-length cDNA sequence of *PxABCH1* with a specific primer set (Table S1) to detect potential sequence divergence in midgut tissues among the susceptible and four Cry1Ac/Btk-resistant *P. xylostella* strains. After large-scale sequencing (about 30 clones from two independent cDNA batches), we conducted multiple sequence comparison and didn’t detect indel, synonymous or non-synonymous mutations in the *PxABCH1* cDNA sequences in all the resistant strains when compared to the susceptible strain, suggesting that sequence mutations in *PxABCH1* have no role in *P. xylostella* Cry1Ac resistance. In addition, based on the full length of *PxABCH1* clones, we found 8, 8, 9, 8 and 7 synonymous SNPs in DBM1Ac-S, DBM1Ac-R, NIL-R, SZ-R and SH-R strains, respectively. Among all the synonymous SNPs, the susceptible and the four resistant strains shared only one SNP, while other SNPs were unique to each of the susceptible and resistant strain (Table S2). Although we found some unique synonymous SNPs in each resistant strain, these SNPs seem to be normal sequence polymorphisms rather than the cause of resistance because only one or two of the 30 clones contained these unique polymorphisms in the resistant strains; if the SNPs were responsible for Cry1Ac resistance, we would expect that all of the clones from each resistant strain should contain these unique non-synonymous SNPs.

**Figure 4:**
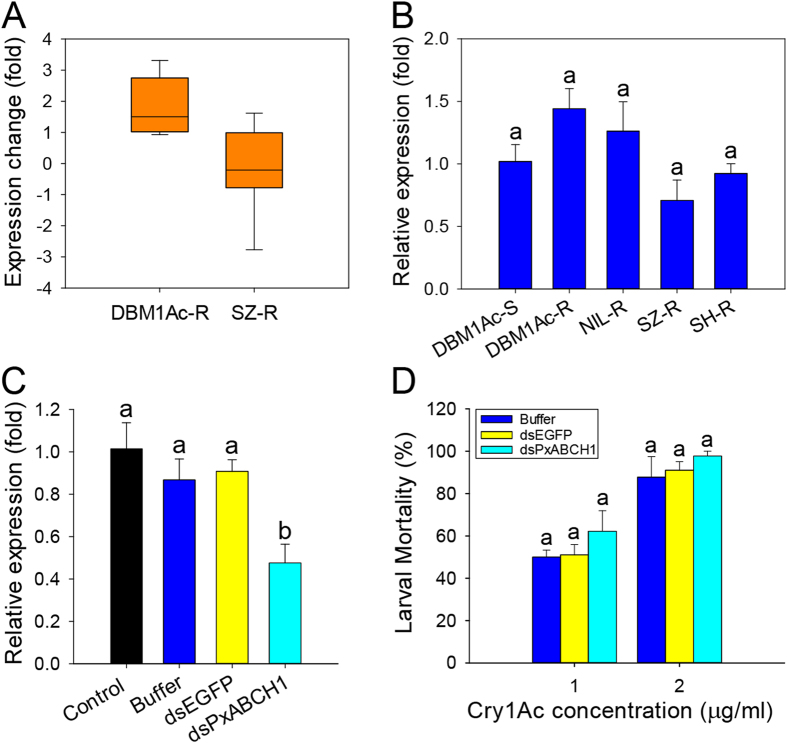
Expression and functional analyses of the *PxABCH1* gene in *P. xylostella*. (**A**) Expression fold changes of *PxABCH1* in two Cry1Ac-resistant *P. xylostella* strains when compared to the susceptible *P. xylostella* strain. The expression levels of *PxABCH1* were calculated based on the RPKM (the number of reads per kb of exon region per million mapped reads) values [log_2_ratio (DBM1Ac-R- or SZ-R-RPKM/DBM1Ac-S-RPKM)] of its unigenes derived from our previous transcriptome and RNA-Seq libraries^38^,^39^. (**B**) Relative expression levels of *PxABCH1* as determined by qPCR in midgut tissues from fourth-instar larvae of all the susceptible and Cry1Ac/Btk-resistant *P. xylostella* strains. Expression of the *RPL32* gene was used as the internal reference gene to normalize data sets and calculate expression levels. The relative expression levels (fold) were calculated by assigning an arbitrary value of 1 to the expression level in the susceptible DBM1Ac-S strain. Values shown in the figure are means and standard errors. Expression values with the same letter are not significantly different based on three biological replications and four technical repeats (P > 0.05; Holm-Sidak’s test; n = 3). (**C**) RNAi-mediated sublethal suppression of *PxABCH1* expression after a low dose of its specific dsRNA (dsPxABCH1) injection at 48 h post-RNAi. The non-injection (control) or injection of *P. xylostella* larvae with buffer and dsEGFP were all used as negative controls. Expression of the *RPL32* gene was used as the internal reference gene. The relative expression levels (fold) were calculated by assigning an arbitrary value of 1 to the expression level in the non-injection (control) DBM1Ac-S larvae. Values shown in the figure are means and standard errors. Different letters indicate significant differences between treatments (P < 0.05; Holm-Sidak’s test; n = 3). (**D**) Susceptibility of DBM1Ac-S larvae to two concentrations of Cry1Ac protoxin as affected by prior injection with buffer, dsEGFP or dsPxABCH1. Within each toxin concentration, the same letter indicate no significant difference between treatments (P > 0.05; Holm-Sidak’s test; n = 3).

After re-analyzing the RPKM values of all the *PxABCH1* unigenes derived from our previous transcriptome and RNA-seq studies^38^,^39^, we found that the *PxABCH1* gene tended to be differentially expressed in the midgut tissue from Cry1Ac-resistant DBM1Ac-R and SZ-R strains when compared to the Cry1Ac-susceptible DBM1Ac-S strain (Fig. 4A). Therefore, to investigate whether an alteration of *PxABCH1* expression is associated with Cry1Ac resistance in *P. xylostella*, we measured *PxABCH1* expression levels in the midgut tissues of fourth-instar larvae from the susceptible and four resistant *P. xylostella* strains (Fig. 4B). The qPCR results showed that *PxABCH1* expression actually did not significantly differ among the susceptible and four resistant strains (P > 0.05; Holm-Sidak’s test; n = 3), suggesting that Cry1Ac resistance in *P. xylostella* is not associated with the expression level of *PxABCH1*.

To further test whether the *PxABCH1* gene correlates with the Cry1Ac resistance in *P. xylostella*, we used RNA interference (RNAi) to silence its expression by injecting a sublethal dose (100 ng) of its dsRNA (dsPxABCH1) into the early third-instar DBM1Ac-S larvae. The dsPxABCH1 sequence was designed to be complementary to the 3′-terminal gene-specific CDS region (nucleotides 1042 to 2081) of the *PxABCH1* mRNA to avoid the potential off-target effect with other two closely related PxABCH genes. Relative *PxABCH1* expression levels in control and treated larvae (non-injected, injected with buffer or dsEGFP) were determined at 48 h post-injection, and injection of dsPxABCH1 into larvae significantly reduced *PxABCH1* transcript levels by about 50% relative to controls (P < 0.05; Holm-Sidak’s test; n = 3) (Fig. 4C). Subsequent bioassays performed at 48 h post-injection demonstrated that silencing of *PxABCH1* gene expression did not significantly altered the larval susceptibility to Cry1Ac protoxin when compared to the controls (P > 0.05; Holm-Sidak’s test; n = 3) (Fig. 4D). The results indicated that *PxABCH1* gene is actually independent of Bt Cry1Ac resistance in *P. xylostella*.

### Effect of silencing the *PxABCH1* gene on larval and pupal mortality

**Figure 5:**
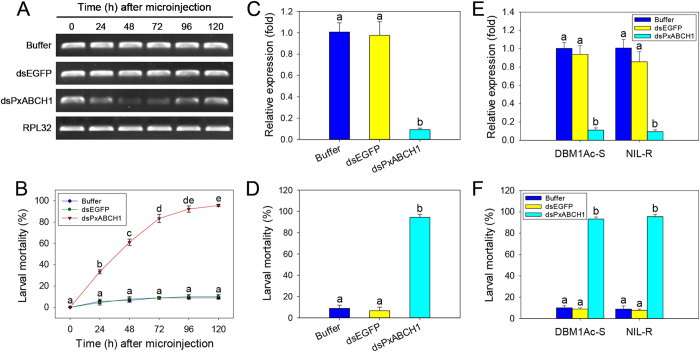
The effect of silencing of *PxABCH1* expression by both injection and oral delivery on the larval mortality of the susceptible DBM1Ac-S and Cry1Ac-resistant NIL-R *P. xylostella* strains. (**A**) Suppression of *PxABCH1* expression in DBM1Ac-S larvae by microinjection was detected by RT-PCR analysis at different times after injection of specific dsRNA. (**B**) Effect of injection with dsPxABCH1 on mortality of DBM1Ac-S larvae at different times post-injection but before pupation. (**C**) Suppression of *PxABCH1* expression in NIL-R larvae after dsRNA injection was detected at 48 h post-injection by qPCR analysis. (**D**) Effect of injection with dsPxABCH1 on the mortality of NIL-R larvae at 120 h after injection but before pupation. (**E**) Suppression of *PxABCH1* expression after dsRNA oral delivery in both DBM1Ac-S and NIL-R larvae was detected at 48 h post-injection by qPCR analysis. (**F**) Effect of oral delivery with dsPxABCH1 on the mortality of both DBM1Ac-S and NIL-R larvae at 120 h after injection but before pupation. Expression of the *RPL32* gene was used as the internal reference gene to confirm the integrity of the cDNA samples or to normalize data sets and calculate expression levels. Buffer- and dsEGFP-injected *P. xylostella* larvae were used as controls. The relative expression levels (fold) were calculated by assigning an arbitrary value of 1 to the expression level in buffer-injected *P. xylostella* larvae. Values shown in the figure are means and standard errors from three biological repeats. Different letters indicate significant differences between treatments (*P* < 0.05; Holm-Sidak’s test; n = 3).

To investigate the effect of silencing the *PxABCH1* gene on larval and pupal mortality, we delivered a relatively high dose of dsRNA (300 ng) into the early third-instar DBM1Ac-S or NIL-R larvae by both injection and oral delivery. For dsRNA microinjection, RT-PCR results showed that *PxABCH1* expression in DBM1Ac-S larvae was significantly decreased at 24 h post-injection and was the lowest at 48 h post-injection, moreover, this remarkable knockdown effect was subsequently maintained for more than 48 h, whereas *PxABCH1* expression levels were not obviously altered in both buffer- and dsEGFP-injected control groups (Fig. 5A). Simultaneously, we found that the larval mortality was much greater following injection of dsPxABCH1 than buffer or dsEGFP (P < 0.05; Holm-Sidak’s test; n = 3) (Fig. 5B). In particular, at 120 h post-injection before pupation, mortality was >95% in the DBM1Ac-S larvae injected with dsPxABCH1 but was <10% in the larvae injected with buffer or dsEGFP post-RNAi (Fig. 5B). Furthermore, similar results were obtained with the NIL-R strain. The qPCR result revealed that injection of NIL-R larvae with dsPxABCH1 greatly reduced *PxABCH1* expression by more than 90% relative to the controls at 48 h post-injection (*P *< 0.05; Holm-Sidak’s test; n = 3) (Fig. 5C) and dramatically increased larval mortality (>94%) relative to the controls (<10%) at 120 h post-injection (P < 0.05; Holm-Sidak’s test; n = 3) (Fig. 5D). For dsRNA oral delivery, likewise, the qPCR result revealed that oral delivery of dsPxABCH1 into DBM1Ac-S or NIL-R larvae can also greatly reduced *PxABCH1* expression by approximately 90% relative to the controls at 48 h post-injection (*P *< 0.05; Holm-Sidak’s test; n = 3) (Fig. 5E) and dramatically increased larval mortality (>93%) relative to the controls (<10%) at 120 h post-injection (P < 0.05; Holm-Sidak’s test; n = 3) (Fig. 5F). In addition, after careful observation, we found that the RNAi conducted by both dsRNA injection and oral delivery induced an unconspicuous larval desiccation phenotype, suggesting that the PxABCH1 may also participate in cuticular lipids transport like its ortholog TcABCH-9C in *T. castaneum*.

Although larval mortality was extremely high following dsPxABCH1 injection or oral delivery, a few dsPxABCH1-treated larvae in both strains can still survive and pupate. However, the survived larvae were obviously unhealthy and didn’t like to move and feed, and we subsequently measured how the injection or oral delivery of larvae with buffer, dsEGFP or dsPxABCH1 affected the pupation rate, pupal weight, and eclosion rate. The statistical results showed that larvae survived from dsPxABCH1-treatment in both strains displayed significantly reduced pupation rate, pupal weight and eclosion rate (0%, all dead) when compared to the controls (P < 0.05; Holm-Sidak’s test; n = 3) (Table 1). These results indicate that silencing *PxABCH1* gene expression by a relatively high dose of dsRNA is lethal to larvae and pupae in both susceptible and Cry1Ac-resistant *P. xylostella*.

## Discussion

Recently, ABC transporters have become a focus of research in arthropods mainly due to their important roles in xenobiotic transport and insecticide resistance, especially for members in the ABCB, ABCC, and ABCG subfamilies^36^,^37^. In contrast, the enigmatic ABCH subfamily members have received relatively little attention, and their functions and applications remain to be explored in arthropods. In this study, the cloned *PxABCH1* in *P. xylostella* is the first characterized ABCH gene in lepidopterans.

Recent reports indicate that the *P. xylostella* genome might contain two to four copies of *PxABCH1* that is orthologous to the *ABCH1* gene *CG9990* in *D. melanogaster*^26^,^37^. Genome scaffold information in the DBM-DB (http://iae.fafu.edu.cn/DBM/search.php) revealed two potential tandem repeat copies of *PxABCH1* juxtaposed in a head-to-tail orientation on each of the two scaffolds (Px014955 and Px014956 on scaffold_677, Px005111 and Px005110 on scaffold_19). Our sequence alignment and genome scaffold analyses, however, revealed that the small scaffold_677 is more likely to be a part of the large scaffold_19 and that the two tandem-repeat ABCH genes in scaffold_677 have high sequence similarity to the two tandem-repeat ABCH genes in scaffold_19 (the genomic sequence quality of scaffold_19 is much higher than scaffold_677); moreover, our further sequence alignment analyses showed that the two tandem-repeat ABCH genes in each scaffold (*PxABCH1* and *PxABCH2*) are not two copies of the same ABCH gene but may be generated by tandem duplication during evolution since that their CDS sequence similarity reaches as high as 85%. Hence, both *PxABCH1* and *PxABCH2* are actually single-copy genes (Px014955 on scaffold_677 or Px005111 on scaffold_19) in the *P. xylostella* genome. In addition, another *PxABCH3* gene (Px003594 on scaffold_158) has also been found in the *P. xylostella* genome. In fact, our findings have already been confirmed by analyzing and comparing them with the recent *P. xylostella* genome data that is relatively well re-annotated by the NCBI (*PxABCH1*: GenBank accession nos. XM_011549433/XM_011549434 or XM_011567668/XM_011567669; *PxABCH2*: GenBank accession nos. XM_011549432 or XM_011567670; *PxABCH3*: GenBank accession no. XM_011557937).

According to a previous study, it is plausible that *ABCH1* gene is closely related to ABCG subfamily members despite the low sequence identity between them^30^. Moreover, the high similarity of protein secondary structure between ABCH and ABCG genes could explain why *ABCH1* in many species is generally falsely annotated as *ABCG20* or *ABCG23* in the GenBank database. Intriguingly, although the protein structure organization is similar for *PxABCH1* and PxABCG genes, our sequence alignment and phylogenetic analysis first show that *PxABCH1* is actually more closely related to ABCA members than to ABCG members. The NCBI CDD-based protein annotation incorrectly denoting PxABCH1 as an ABCA member also confirms the high sequence similarity between ABCH and ABCA members. This finding was also confirmed by the phylogenetic analyses of the whole ABC transporter superfamily in the *Bombyx mori*^24^, *P. xylostella*^26^, *T. castaneum*^35^, *T. urticae*^28^ and *Chrysomela populi*^46^.

Determination of tissue- and stage-specific expression showed that *PxABCH1* is widely expressed in five different *P. xylostella* tissues and all the developmental stages with the highest expression in the head and the male adult. In the zebrafish *D. rerio*, *DrABCH1* expression is high in the brain, gills, and kidney and low in the intestine, gonads, skeletal muscle and liver^30^. In the silkworm *B. mori*, *BmABCH2* expression can be detected in all the 10 tested tissues^25^. In the fruit fly *D. melanogaster*, the ABCH gene *CG9990* expressed in diverse tissues and enriched in the adult crop and hindgut^47^. In the red flour beetle *T. castaneum*, *TcABCH-9C* expression can be detected in all developmental stages and is highest in pre-pupae^35^. In the spider mite *T. urticae*, *TuABCH1* (Tetur ID: tetur01g05970) is also highly expressed in all developmental stages^28^. In the western tarnished plant bug *L. hesperus*, the expression of *LhABCH1* gene were detected in all the developmental stages and tissues/segments in both sexes^41^. In the phytophagous leaf beetle *C. populi*, all the three putative ABCH genes were low expressed in the larval gut and Malpighian tubules, and two of them were relatively high expressed in the fat body and glands^46^. The wide expression of *ABCH1* and other ABCH genes in these arthropods indicates they are versatile genes that can be involved in many basic physiological functions in different developmental stages and tissues. Considering that both ABCA and ABCG members, which are closely related to ABCH1, play important roles in lipid transport in mammals^19^, we speculated that ABCH1 might also be responsible for lipid transport in arthropods. Recently, an excellent study utilized RNAi to knockdown multiple ABC transporter genes including *TcABCG-4C* and *TcABCH-9C* in both larvae and adults of *T. castaneum*^34^. Knockdown of *TcABCG-4C* gene caused larval desiccation phenotype, reduced egg numbers and failed egg hatching, and further detection showed that knockdown of *TcABCG-4C* gene can result in reduced lipid staining in the *T. castaneum* epicuticle, suggesting it might be involved in the transport of cuticular lipids^34^. Intriguingly, knockdown of *TcABCH-9C* gene showed the similar larval desiccation phenotype and negative effects on adult egg-laying and egg-development to *TcABCG-4C* gene, suggesting that TcABCH-9C might also act as cuticular lipid transporters^34^. In this study, we also conducted RNAi with a relatively high dose of dsRNA to knockdown the *PxABCH1* expression in *P. xylostella* larvae, and we also obtain an unconspicuous larval desiccation phenotype and 100% larval and pupal mortality before reaching the adult stage, suggesting that PxABCH1 may also act as transporters of cuticular lipids like its ortholog TcABCH-9C in *T. castaneum*. Of course, we can speculate that PxABCH1 may also have other important physiological functions. For example, the high expression of *PxABCH1* gene in head and the male adult may indicate that it can also participate in regulation and maintenance of lipid homeostasis just like ABCA and ABCG members in mouse brain^48^ and participate in male sexual maturation or spermatozoa protection, further studies are merited to determine whether it can be involved in these important physiological processes. Moreover, microarray and RNA-Seq expression data have shown increased expression of ABCH genes in two arthropods resistant to different insecticides including *P. xylostella*^26^ and *T. urticae*^37^, suggesting that arthropod ABCH genes might also be associated with insecticide transport and resistance. In addition, increased expression of ABCH genes might also be involved in other physiological processes such as cold tolerance in *D. melanogaster*^49^ and diapause in *T. urticae*^50^.

Although RNAi technology is promising for the control of insect pests, its use faces many challenges^11^,^51^. The effect of RNAi-mediated gene silencing differs among insect species, and may be difficult to achieve especially in lepidopterans^52^. However, *P. xylostella* may be an exception because diverse functional genes in its larval midgut and even other tissues can be silenced either by microinjection or oral delivery^14^,^15^,^16^,^17^,^53^,^54^. While injection of dsRNA or siRNA is not practical for *P. xylostella* control, oral delivery of dsRNA or siRNA should be feasible. Of particular note, the most promising RNAi-based method for control of *P. xylostella* probably involves the plant-mediated knockdown of specific genes^55^,^56^.

The selection of suitable target gene is of great significance to the RNAi-based insect control strategy. In general, functional genes encoding essential proteins can be suitable RNAi targets as part of arthropod pest control strategies. Given that the *ABCH1* gene has diverse and essential physiological functions in phylogenetically distant arthropod species, it should be an effective target for RNAi-based control of diverse insect pests. Moreover, since that the *ABCH1* gene is not present in vertebrates except for teleosts, it will greatly reduce the possibility of non-target effects and considerably reinforce the biosafty to higher organisms. Regarding the *PxABCH1* gene in *P. xylostella*, the dsRNA can be designed in the 3′-terminal gene-specific TMD region to avoid potential intergenic off-target effects and cross-species non-target effects. Moreover, we found that the cDNA sequence of *PxABCH1* is not polymorphic in the same or even different *P. xylostella* strains, which will apparently reduce the possibility of RNAi resistance development.

In this study, we achieved similar high silencing efficacy of *PxABCH1* gene by both injection and oral delivery of its gene-specific dsRNA into *P. xylostella* larvae, both can result in nearly 100% larval mortality and the few survived *P. xylostella* larvae subsequently died as pre-pupae or pupae. Likewise, the knockout mutants and a knockdown RNAi line of its orthologous gene *CG9990* in *D. melanogaster* are both lethal^34^, while *TcABCH-9C* gene silencing in *T. castaneum* can result in 100% mortality in injected larvae and the complete hatching failure of eggs produced by injected adults^35^. Interestingly, the rapid and high larval mortality resulted from RNAi of *PxABCH1* and its orthologous genes would be used as an indicator for confirming the efficiency of RNAi in these insects. More importantly, we found that *PxABCH1* is not associated with Bt Cry1Ac resistance and that RNAi injection can cause 100% larval and pupal mortality in a Bt Cry1Ac-resistant strain of *P. xylostella*, suggesting that this gene is an excellent RNAi target for the control of both susceptible and Bt-resistant *P. xylostella*. Of peculiar interest, *PxABCH1* was found to be the most up-regulated ABC transporter gene in two insecticide-resistant *P. xylostella* strains^26^, together with previous reports showed that significantly increased ABCH gene expression levels in other insecticide-resistant arthropods such as *T. urticae*^37^, we speculate that silencing of *PxABCH1* orthologous genes can also lead to significantly reduced insecticide resistance and an extremely low survival rate in these arthropods. Therefore, ABCH1 might be an excellent target for RNAi-based insect control and Bt/chemical insecticide resistance management. Considering that transgenic corn hybrids expressing the insect-killing V-ATPase dsRNA and the corn rootworm-active Bt protein Cry3Bb1 provide better root protection than hybrids expressing either Bt Cry3Bb1 or V-ATPase dsRNA alone^11^, further research is warranted on the use the ABCH1 as a RNAi target in other Bt/chemical insecticide-resistant arthropods, and it will be very important to develop the next-generation insect-resistant transgenic crops that combine Bt- and RNAi-based insect control technologies (Bt + RNAi strategy) as a pivotal part of the IPM programs.

## Methods

### Insect strains

The five susceptible and Bt-resistant *P. xylostella* strains used in this study have been described in detail elsewhere^57^. Briefly, the susceptible *P. xylostella* strain DBM1Ac-S (originated from Geneva, NY, USA, also called Geneva 88) and the Cry1Ac-resistant DBM1Ac-R (originated from Loxahatchee, Florida, USA, also called Cry1Ac-R) was originally provided by Drs. J. Z. Zhao and A. Shelton (Cornell University, USA) in 2003. The Cry1Ac-resistant *P. xylostella* strain NIL-R, which is near-isogenic to the susceptible DBM1Ac-S strain, was recently constructed by multiple (six times) backcrossing DBM1Ac-R with DBM1Ac-S followed by the Cry1Ac selection of their offspring^58^. The Cry1Ac-resistant SZ-R and Btk (Bt var. *kurstaki*)-resistant SH-R strains originated from moths collected in China at Shenzhen (2003) and Shanghai (2005), respectively. The susceptible DBM1Ac-S strain was kept unselected and without exposure to any Bt toxins or chemical insecticides, while all the resistant strains were kept under constant selection with Cry1Ac protoxin solution or Btk formulation that regularly kills 50–70% of the larvae on sprayed cabbage leaves. All the *P. xylostella* strains were reared on JingFeng No. 1 cabbage (*Brassica oleracea* var. *capitata*) at 25 °C, 65% RH, and a 16D:8L photoperiod. Adults were fed with a 10% sucrose solution.

### Preparation of Cry1Ac protoxin and bioassay

The Cry1Ac protoxin was extracted and purified from Bt var. *kurstaki* strain HD-73 by isoelectric point precipitation as previously described^59^, and the purified Cry1Ac protoxin was subsequently quantified by densitometry.

To determine the resistance ratio of all the resistant *P. xylostella* strains, a leaf-dip bioassay was conducted as described elsewhere^57^. The final bioassay results showed that the resistance ratios (resistant larvae LC_50_ value divided by susceptible larvae LC_50_ value) of the DBM1Ac-R, NIL-R and SZ-R strains to Cry1Ac protoxin was about 3500-, 4000-, and 450-fold compared to the DBM1Ac-S, respectively, and the resistance ratio of SH-R strain to a Btk formulation (WP with potency of 16,000 IU/mg, provided by Bt Research and Development Centre, Agriculture Science Academy of Hubei Province, China) was about 1900-fold compared to the DBM1Ac-S strain.

### RNA extraction and cDNA synthesis

Total RNA was extracted from different samples of all the *P. xylostella* strains using TRIzol reagent (Invitrogen, Carlsbad, CA, USA) according to the manufacturer’s instructions. RNA integrity was determined using agarose gel electrophoresis and RNA was quantified with a NanoDrop 2000c spectrophotometer (Thermo Fisher Scientific Inc., Waltham, MA, USA). For gene cloning, the first-strand cDNA was prepared using 5 μg of total RNA with the PrimeScript^TM^ II 1st strand cDNA Synthesis Kit (TaKaRa, Dalian, China) following the manufacturer’s recommendations. For gene expression analysis, the first-strand cDNA was prepared using 1 μg of total RNA with the PrimeScript RT kit (containing gDNA Eraser, Perfect Real Time) (TaKaRa, Dalian, China) following the manufacturer’s recommendations. The synthesized first-strand cDNA was used immediately or was stored at −20 °C until used.

### Gene cloning and sequence variation detection

For *PxABCH1* gene cloning, one unigene (Fig. S1, Fragment 1,636 bp from nucleotides 151 to 786) was obtained from our previous *P. xylostella* transcriptome database^38^. The PCR cloning strategies and the gene-specific primers designed to obtain overlapping PCR amplicons are shown in Fig. S1 and Table S1, respectively. To obtain the full-length cDNA sequence of the *PxABCH1* gene, 3′, 5′-rapid amplification of cDNA ends (RACE) was performed with SMARTer^TM^ RACE cDNA Amplification kits (Clontech, Mountain View, CA, USA) using the midgut cDNA samples from the fourth-instar larvae as template following the manufacturer’s protocols. The full-length cDNA of *PxABCH1* was obtained by overlapping and assembling these cDNA fragments, and then the coding sequence was validated by PCR amplification using a specific primer set. According to the guidelines set forth by the HUGO gene nomenclature committee (HGNC), we classify this cloned ABC transporter gene in the H subfamily. The full-length *PxABCH1* cDNA sequence has been deposited in the GenBank database under accession number KP260785. Large-scale sequencing and comparison of the full-length *PxABCH1* cDNA in the midgut tissues were performed to detect potential sequence variations among the susceptible DBM1Ac-S and all the four resistant strains.

PCR reactions were performed in an S1000^TM^ Thermal Cycler PCR system (BioRad, USA) for 35 cycles, each consisting of denaturing at 94 °C for 30 s, annealing at 50–60 °C (depending on the primer) for 45 s, and extension at 72 °C for 1–3 min based on the product size, followed by a final extension of 10 min at 72 °C using LA Taq polymerase (TaKaRa, Dalian, China). All of the cloning primers for each gene were designed with Primer Premier 5.0 software (Premier Biosoft, Canada). Amplicons of the expected size were excised from 1.5–2.0% agarose gels, purified using the TIANgel Midi Purification Kit (TIANGEN, Beijing, China), and subcloned into the pEASY-T1 vector (Transgen, Beijing, China) before transformation into *Escherichia coli* TOP10 competent cells (Transgen, Beijing, China) for sequencing.

### *In silico* gene sequence analysis

Gene sequence was assembled and multiple sequences were aligned with DNAMAN 7.0 (Lynnon BioSoft, USA). The open reading frame of the target nucleotide sequence was found using the ORF Finder at the NCBI website (http://www.ncbi.nlm.nih.gov/gorf/gorf.html). The nucleotide sequence-similarity analyses were performed using BLAST at the NCBI website (http://blast.ncbi.nlm.nih.gov/). The deduced protein sequence was obtained with the ExPASy translation tool Translate (http://web.expasy.org/translate/). The isoelectric point (pI) and molecular weight (Mw) were calculated with the ExPASy proteomics tool Compute pI/Mw (http://ca.expasy.org/tools/pi_tool.html). The N-terminal signal peptide was determined using the SignalP 4.0 server (http://www.cbs.dtu.dk/services/SignalP/). The transmembrane domain (TMD) and membrane topology was analyzed with TOPCONS online software (http://topcons.cbr.su.se/). The nucleotide-binding domain (NBD) was annotated with the ScanProsite software (http://prosite.expasy.org/scanprosite/) with the Prosite domain profile PS50893. The presence of N- and O-glycosylation sites on the predicted protein sequence was tested using the NetNGlyc 1.0 (http://www.cbs.dtu.dk/services/NetNGlyc/) and NetOGlyc 4.0 server (http://www.cbs.dtu.dk/services/NetOGlyc/), respectively.

### Phylogenetic tree construction

Most recently, the whole diamondback moth genome has been relatively well re-annotated by the NCBI (National Center of Biotechnology Information) and have been deposited in the GenBank database (http://www.ncbi.nlm.nih.gov/). To confirm our cloned ABC transporter gene does belong to the ABCH subfamily and to determine the phylogenetic relationship between the ABCH subfamily and other closely related ABC transporter subfamilies, we extracted and eliminated the redundant amino acid sequences of all the putative ABCA, ABCG and ABCH genes in the *P. xylostella* genome from the GenBank database, and the identified NBDs of these ABC transporters by ScanProsite software were used to construct the phylogenetic tree. Furthermore, to see the phylogenetic relationship of this gene among different organisms, full-length amino acid sequences of *PxABCH1* orthologous genes in diverse species were retrieved from GenBank or *T. urticae* genome database (http://metazoa.ensembl.org/Tetranychus_urticae/Info/Index) to construct another phylogenetic tree. Amino acid sequences were first aligned with ClustalW using Molecular Evolutionary Genetic Analysis software version 6.0 (MEGA 6) (http://www.megasoftware.net/). The phylogenetic tree was then constructed using the neighbor-joining (NJ) method with “p-distance” as the amino acid substitution model, “pairwise deletion” as the gaps/missing data treatment and 1000 bootstrap replications.

### Sample preparation

To determine where the *PxABCH1* gene is expressed, five tissues (head, integument, midgut, testis and Malpighian tubules) were dissected from fourth-instar DBM1Ac-S larvae. To determine whether *PxABCH1* is differentially expressed during *P. xylostella* development, we analyzed samples from eggs, first- to fourth-instar larvae, prepupae, pupae, 1-day-old pre-copulatory males and virgin adult females. To detect the potential sequence variations or expression differences among all the susceptible and Bt Cry1Ac/Btk-resistant *P. xylostella* larvae, midgut tissues were dissected from fourth-instar larvae of all the five tested *P. xylostella* strains. Total RNA was isolated from these samples and cDNA was synthesized as described above.

### Quantitative PCR (qPCR) analysis

The qPCR technique was used to quantify *PxABCH1* expression. A gene-specific primer set for *PxABCH1* was designed and used in the PCR reaction. The reaction volume (25 μl) contained 9.5 μl of ddH_2_O, 12.5 μl of 2× SuperReal PreMix Plus (TIANGEN, Beijing, China), 7.5 μM of each specific primer, 11 ng of first-strand cDNA template, and 0.5 μl of 50× ROX Reference Dye (TIANGEN, Beijing, China). The qPCR program included an initial denaturation for 15 min at 95 °C followed by 40 cycles of denaturation at 95 °C for 15 s, annealing for 30 s at 60 °C, and extension for 32 s at 72 °C. For melting curve analysis, an automatic dissociation step cycle was added. Reactions were performed in an ABI 7500 Real-Time PCR system (Applied Biosystems, USA) with data collection at stage 2, step 3 in each cycle. Amplification efficiencies and linear correlation between the quantity of cDNA template and the quantity of PCR product generated by the gene-specific primers were calculated from the dissociation curve of four replicates using five 2-fold serial dilutions (1:1, 1:2, 1:4, 1:8, and 1:16). Only results with single peaks in melting curve analyses, 95–100% primer amplification efficiencies, and >0.95 correlation coefficients were used for subsequent data analysis. For negative control reactions, cDNA template was replaced with ddH_2_O, which resulted in no amplified products. The amplified fragments were sequenced to confirm that potential expression differences were not due to sequence variations in the targeted genes. Relative quantification was performed using the 2^−ΔΔCt^ method^60^, and data were normalized to the ribosomal protein *L32* (*RPL32*) gene (GenBank accession no. AB180441) as validated elsewhere^15^,^26^. Four technical replicates and three biological replicates were used for each treatment. One-way ANOVA with Holm-Sidak’s tests (overall significance level = 0.05) was used to determine the significant statistical difference between treatments.

### RNA interference

RNA interference (RNAi)-mediated knockdown of *PxABCH1* expression was conducted by microinjection or oral delivery of its dsRNA into early third-instar *P. xylostella* larvae to determine whether *PxABCH1* is involved in *P. xylostella* Cry1Ac resistance and to confirm the lethal effect of its relatively high dose of dsRNA to both Cry1Ac-susceptible and resistant larvae. Specific dsRNA primers containing a T7 promoter sequence at the 5′ end targeting the *PxABCH1* (GenBank accession no. KP260785) or *EGFP* (GenBank accession no. KC896843) were designed (Table S1) using the Primer Premier 5.0 software (Premier Biosoft, Canada). The primer set used to generate dsRNA of *PxABCH1* was designed to the 3′-terminal gene-specific TMD region and not in the intergenic conserved NBD region to avoid potential off-target effects. After amplified from midgut cDNA samples of *P. xylostella* larvae and confirmed by sequencing, the amplicons (492 bp for dsPxABCH1 and 469 bp for dsEGFP) were used as template for *in vitro* transcription reactions to generate dsRNAs using the T7 Ribomax^TM^ Express RNAi System (Promega, Madison, WI, USA). After the synthesized dsRNAs were suspended in a buffer solution [10 mM Tris–HCl (pH 7.0); 1 mM EDTA], the preparation was subjected to 1% agarose gel electrophoresis and the dsRNA was then quantified spectrophotometrically. To increase dsRNA stability and facilitate dsRNA delivery, buffer or dsRNA solution was mixed with the Metafectene PRO transfection reagent (Biontex, Planegg, Germany) before microinjection in a 1:1 volume ratio and incubated for 20 min at 25 °C. The detection of silencing effect and the quantities of dsRNA injected were optimized in preliminary experiments.

Early third-instar larvae were microinjected with dsRNA with the aid of an SZX10 microscope (Olympus, Tokyo, Japan). The Nanoliter 2000 microinjection system (World Precision Instruments Inc., Sarasota, FL, USA) with sterilized fine glass capillary microinjection needles pulled by a P-97 micropipette puller (Sutter Instrument, Novato, CA, USA) was used to deliver 70 nanoliters of injection buffer or dsRNAs (both containing the Metafectene PRO solution) into the hemocoel of early third-instar *P. xylostella* larvae. The volume of sample microinjected into each larva resulted in <10% larval mortality 5 days post-injection. Larvae were starved for 6 h and cold-anesthetized for 30 min on ice before microinjection. More than 30 larvae were injected for each treatment, and three independent experiments performed. Injected larvae were allowed to recover for about 3 h at room temperature and were then returned to standard rearing conditions for the subsequent experiments.

To determine whether *PxABCH1* is involved in *P. xylostella* Cry1Ac resistance, 70 nl of the injection buffer or 100 ng dsRNAs (both containing the Metafectene PRO solution) was delivered into the hemocoel of the early third-instar DBM1Ac-S *P. xylostella* larvae. The RNAi effectiveness was then confirmed by qPCR at 48 h post-injection using cDNA prepared from isolated total midgut RNA, and the qPCR conditions were as described above. Leaf-dip bioassays were subsequently performed for 72 h using larvae at 48 h after dsRNA injection and two Cry1Ac protoxin concentrations (1 and 2 μg**/**ml, approximated the respective LC_50_ and LC_90_ value for non-injected DBM1Ac-S larvae). Bioassays were performed with 30 larvae per RNAi treatment and toxin concentration, and each bioassay was replicated three times. Mortality in control treatments was <5%, and bioassay data processing was as described elsewhere^57^. One-way ANOVAs with Holm-Sidak’s tests (overall significance level = 0.05) were used to determine the differences between qPCR and toxin bioassay treatments.

In addition, to investigate the lethal effect of the relatively high dose of *PxABCH1* dsRNA, we conducted both the injection and oral delivery of dsRNA into the early third-instar *P. xylostella* larvae to detect the mortality. For dsRNA microinjection, 70 nl of the injection buffer or 300 ng dsRNAs (both containing the Metafectene PRO solution) was delivered into hemocoel of the early third-instar DBM1Ac-S and NIL-R larvae, the subsequent experimental process is as mentioned above. Meanwhile, a dsRNA droplet feeding method^15^ was also adopted to conduct the RNAi experiment. The early third-instar DBM1Ac-S and NIL-R larvae were initially starved for 24 h, and then they were droplet-fed 0.14 microliter of oral delivery buffer or oral delivery buffer containing 300 ng of dsRNAs (both containing the Metafectene PRO solution) using an ordinary pipettor (Eppendorf, Hamburg, Genmany). More than 30 larvae were fed for each treatment, and three independent experiments performed. The droplet-fed larvae were allowed to recover for about 3 h at room temperature and were then returned to standard rearing conditions for the subsequent experiments. For dsRNA microinjection, RNAi effectiveness in DBM1Ac-S larvae was assessed by RT-PCR at 24-h intervals from 0 to 120  h post-injection and the RNAi effectiveness in NIL-R larvae was assessed by qPCR at 48 h post-injection. For dsRNA oral delivery, the RNAi effectiveness in both DBM1Ac-S and NIL-R larvae was assessed by qPCR at 48 h post-feeding. In both cases, cDNA prepared from total isolated larval RNA was used, and RT-PCR and qPCR conditions were the same as the qPCR conditions described above. For dsRNA microinjection, larval mortality was recorded in the control and treatment groups every 24 h for 5 days for DBM1Ac-S larvae and after 120 h for NIL-R larvae. For dsRNA oral delivery, larval mortality was recorded in the control and treatment groups after 120 h for both DBM1Ac-S and NIL-R larvae. Furthermore, RNAi effects on subsequent developmental stages of surviving larvae of both strains were also analyzed by comparing pupation percentage, pupal weight, and eclosion percentage. Larvae injected with buffer and dsEGFP were used as negative controls, and all the larvae used in the test were fed on fresh cabbage leaves. Each treatment was replicated three times, and one-way ANOVAs with Holm-Sidak’s tests (overall significance level = 0.05) were used to assess the differences between control and treated groups in terms of gene expression, larval mortality, pupation percentage, pupal weight, and eclosion percentage.

## Additional Information

**How to cite this article**: Guo, Z. *et al.* The novel ABC transporter ABCH1 is a potential target for RNAi-based insect pest control and resistance management. *Sci. Rep.* **5**, 13728; doi: 10.1038/srep13728 (2015).

## Supplementary Material

Supplementary Information

## Figures and Tables

**Table 1:** Effect of silencing of the *PxABCH1* gene on some biological parameters of the surviving larvae in *P. xylostella* strain DBM1Ac-S and NIL-R.

Methods	Strain	Treatment	Pupation rate (%)	Pupal weight (mg)	Eclosion rate (%)
Microinjection	DBM1Ac-S	Buffer	100.00 ± 0.00^a^	5.12 ± 0.11^a^	88.89 ± 11.11^a^
		dsEGFP	100.00 ± 0.00^a^	5.03 ± 0.14^a^	100.00 ± 0.00^a^
		dsPxABCH1	55.56 ± 11.11^b^	3.70 ± 0.20^b^	0.00 ± 0.00^b^
	NIL-R	Buffer	100.00 ± 0.00^a^	5.06 ± 0.11^a^	100.00 ± 0.00^a^
		dsEGFP	100.00 ± 0.00^a^	5.10 ± 0.05^a^	88.89 ± 11.11^a^
		dsPxABCH1	44.44 ± 11.11^b^	3.82 ± 0.09^b^	0.00 ± 0.00^b^
Oral delivery	DBM1Ac-S	Buffer	100.00 ± 0.00^a^	5.08 ± 0.15^a^	100.00 ± 0.00^a^
		dsEGFP	100.00 ± 0.00^a^	5.12 ± 0.09^a^	100.00 ± 0.00^a^
		dsPxABCH1	44.44 ± 11.11^b^	3.82 ± 0.12^b^	0.00 ± 0.00^b^
	NIL-R	Buffer	100.00 ± 0.00^a^	5.04 ± 0.12^a^	88.89 ± 11.11^a^
		dsEGFP	100.00 ± 0.00^a^	5.07 ± 0.10^a^	100.00 ± 0.00^a^
		dsPxABCH1	55.56 ± 11.11^b^	3.75 ± 0.07^b^	0.00 ± 0.00^b^

Values shown in the table are means and standard errors, and values with different letters in a column for each strain indicated significant differences as determined by Holm-Sidak’s test (P < 0.05; n = 3).
