# Supplementary material for: The novel ABC transporter ABCH1 is a potential target for RNAi-based insect pest control and resistance management
Source: Sci Rep. 2015 Sep 3;5:13728. doi: 10.1038/srep13728 (PMC4558546; doi:10.1038/srep13728)
Supplement: Supplementary Information [file srep13728-s1.doc]

**Supplementary information**

The novel ABC transporter ABCH1 is a potential target for RNAi-based insect pest control and resistance management

Zhaojiang Guo, Shi Kang, Xun Zhu, Jixing Xia, Qingjun Wu, Shaoli Wang, Wen Xie & Youjun Zhang

Department of Plant Protection, Institute of Vegetables and Flowers, Chinese Academy of Agricultural Sciences, Beijing 100081, China.

**Correspondence:**

Prof. Youjun Zhang, Department of Plant Protection, Institute of Vegetables and Flowers, Chinese Academy of Agricultural Sciences, Beijing 100081, China.

Tel: +86-10-82109518

Fax: +86-10-82109518

E-mail: zhangyoujun@caas.cn

**Figure S1.** A schematic diagram of the strategy used to amplify the full-length cDNA sequence of the *PxABCH1* gene. The structure of the full-length cDNA sequence of *PxABCH1* is shown. Lower lines below the full-length cDNA sequence stand for each of the PCR fragments mentioned in Table S1. Fragment 1 was obtained from our previous transcriptome database.

**Table S1.** List of primers used for studying PxABCH1

| Purpose | Primer name | Primer sequence (5′-3′) | PCR product size (bp) | Position (bp)^a^ |
| --- | --- | --- | --- | --- |
| 3′, 5′-RACE | 3′-Outer nested primer | CAAGACCGTCATCATCACCAC | 2722 | 726–3447 |
|  | 3′-Inner primer 1 | CCAGGCGCACACCATAGGTC |  |  |
|  | 3′-Inner primer 2 | GCAGTGGTATCAACGCAGAGTAC |  |  |
|  | 3′-RACE Oligo dT primer | GCAGTGGTATCAACGCAGAGTAC(T)_26_VN |  |  |
|  | 5′-RACE Oligo dG primer | GGCCACGCGTCGACTAGTAC(G)_14_ | 556 | -110–446 |
|  | 5′-Inner primer 1 | GGCCACGCGTCGACTAGTAC |  |  |
|  | 5′-Inner primer 2 | CCGAAGATCCAGCCGAAGTAC |  |  |
|  | 5′-Outer nested primer | GCTGGGCAGGTCGAGGAAGT |  |  |
| Gene cloning | CH1-Full-F | GTGCTTGTGCCGAGGAAGTGC | 2362 | -49–2313 |
|  | CH1-Full-R | TTATCCTTTCTTGAACCTTAGAACTGTG |  |  |
| qPCR analysis | qCH1-F | TGGTGTTCGGCGTCAAGTGT | 129 | 1928–2056 |
|  | qCH1-R | GGATCGCGTTTCGTTCCAGTT |  |  |
|  | qL32-F | CCAATTTACCGCCCTACC | 120 | — |
|  | qL32-R | TACCCTGTTGTCAATACCTCT |  |  |
| dsRNA synthesis^b^ | dsCH1-F | T7-TGCGACTACAACCCGAAGG | 492 | 1642–2081 |
|  | dsCH1-R | T7-GGGTAGAATGAGCCGAGTGC |  |  |
|  | dsEGFP-F | T7-CCACAAGTTCAGCGTGTCCG | 469 | — |
|  | dsEGFP-R | T7-AAGTTCACCTTGATGCCGTTC |  |  |

^a^Positions corresponding to the full-length cDNA sequence of *PxABCH1* (GenBank accession no. KP260785).

^b^Forward and reverse primers to synthesize dsRNA template used for RNAi assay have T7 RNA polymerase promoter sequence (5′-TAATACGACTCACTATAGGGAGA-3′) appended to their 5′ ends.

**Table S2.** Synonymous and non-synonymous single nucleotide polymorphisms of *PxABCH1* cDNA in all susceptible and resistant *P. xylostella* strains

| **Strains** | **Single nucleotide polymorphism sites** | | | | | | | | |
| --- | --- | --- | --- | --- | --- | --- | --- | --- | --- |
|  | 1 | 2 | 3 | 4 | 5 | 6 | 7 | 8 | 9 |
| **DBM1Ac-S** | | | | | | | | | |
| nt | G/A | T/G | G/A | G/A | G/A | T/C | C/G | A/G |  |
| Position^a^ | 120 | 744 | 816 | 954 | 1422 | 1746 | 1938 | 2061 |  |
| aa | — | — | — | — | — | — | — | — |  |
| Position^a^ | 40 | 248 | 272 | 318 | 474 | 582 | 646 | 687 |  |
| **DBM1Ac-R** | | | | | | | | | |
| nt | T/A | C/T | C/T | T/G | G/A | C/T | C/G | C/G |  |
| Position^a^ | 76 | 132 | 537 | 744 | 816 | 1167 | 1581 | 1938 |  |
| aa | S/T | — | — | — | — | — | — | — |  |
| Position^a^ | 26 | 44 | 179 | 248 | 272 | 389 | 527 | 646 |  |
| **NIL-R** | | | | | | | | | |
| nt | G/A | T/A | G/A | G/A | C/G | C/T | C/T | C/G | A/G |
| Position^a^ | 12 | 543 | 816 | 1422 | 1581 | 1842 | 1920 | 1938 | 2061 |
| aa | — | — | — | — | — | — | — | — | — |
| Position^a^ | 4 | 181 | 272 | 474 | 527 | 614 | 640 | 646 | 687 |
| **SZ-R** | | | | | | | | | |
| nt | A/G | C/T | C/A | T/G | G/C | C/T | G/A | C/G |  |
| Position^a^ | 120 | 144 | 363 | 744 | 1119 | 1311 | 1422 | 1938 |  |
| aa | — | — | — | — | — | — | — | — |  |
| Position^a^ | 40 | 48 | 121 | 248 | 373 | 437 | 474 | 646 |  |
| **SH-R** | | | | | | | | | |
| nt | A/G | T/A | T/G | C/T | G/A | T/C | C/G |  |  |
| Position^a^ | 120 | 543 | 744 | 1167 | 1422 | 1746 | 1938 |  |  |
| aa | — | — | — | — | — | — | — |  |  |
| Position^a^ | 40 | 181 | 248 | 389 | 474 | 582 | 646 |  |  |

^a^Positions corresponding to the full-length cDNA and protein sequences of *PxABCH1* gene, respectively (GenBank accession no. KP260785).
